# Pseudo-duplication of the Gallbladder

**DOI:** 10.5811/cpcem.2019.9.43332

**Published:** 2019-11-19

**Authors:** Jamie Adamski, Divya Mohan, Christopher Waasdorp

**Affiliations:** * Jefferson Northeast, Department of Emergency Medicine, Philadelphia, Pennsylvania; † Jefferson Northeast, Department of Family Medicine, Philadelphia, Pennsylvania

## Abstract

Phrygian cap and its rare relative, pseudo-duplication of the gallbladder, are two radiologic findings that may be revealed on ultrasound evaluation. Correct identification of Phrygian cap and pseudo-duplication should trigger a careful survey of the gallbladder in its entirety to rule out pathology. These anatomic variants may lead to partial under-distension of the gallbladder and can cause the gallbladder wall to appear falsely thickened. Asymptomatic patients with this finding may be safely discharged while symptomatic patients may require further surgical consultation.

## CASE PRESENTATION

**Image 1:**
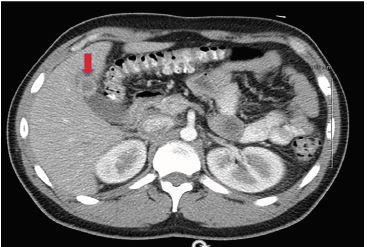
Computed tomography demonstrating gallbladder wall thickening (red arrow).

**Image 2:**
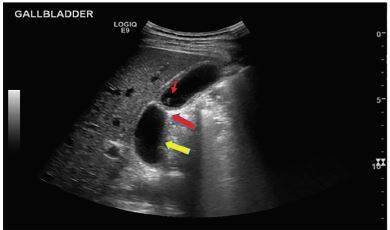
Ultrasound showing a Phrygian cap (large red arrow) and pseudo-duplication (large yellow arrow) with mild gallbladder wall thickening (measuring 3.3 millimeters) and gallstones (small arrow).

A 30-year-old male with history of cholelithiasis presented with right upper and lower quadrant abdominal pain, nausea, vomiting, and subjective fevers. He denied diarrhea, hematochezia, melena, dysuria, hematuria, urinary frequency, chest pain, or shortness of breath. History raised suspicion for cholecystitis versus appendicitis. Labs revealed a mild leukocytosis. Computed tomography showed gallbladder wall thickening (Image 1), and point-of-care ultrasound (Image 2) demonstrated a Phrygian cap with pseudo-duplication of the gallbladder. After surgical consultation, cholescintigraphy was negative for cholecystitis. With successful pain control and oral fluid challenge, the patient was discharged with outpatient surgical follow-up.

## DISCUSSION

Correct identification of Phrygian cap and its rare relative, pseudo-duplication of the gallbladder, warrants careful survey of the gallbladder to rule out underlying pathology.^2^ The term Phrygian cap refers to a portion of the gallbladder that contains an outpouching or folded-over portion. Pseudo-duplication of the gallbladder, a congenital abnormality with an incidence of 1:4000, refers to a duplicate appearance of the gallbladder in the presence of a Phrygian cap. Pseudo-duplication of the gallbladder is associated with congenital biliary obstruction and is important to identify, as the distal segment past the “fold” of the Phrygian cap may be relatively under-distended. This under-distension may allow the gallbladder wall to appear falsely thickened. In an asymptomatic patient, Phrygian cap has no pathological significance and prophylactic cholecystectomy is not necessary. However, in symptomatic patients, further evaluation and surgical consultation may be indicated.^4^ Correct identification of these anatomic variants is important to avoid misidentifying a thickened wall as pathologic in an otherwise normal gallbladder.^3^,^4^

**CPC-EM Capsule**

What do we already know about this clinical entity?

In a patient with right upper quadrant pain, it is important to distinguish a diseased gallbladder from a healthy one.

What is the major impact of the image(s)?

Correct identification of two anatomic variants in the gallbladder, Phyrgian cap and pseudo-duplication, will help determine whether or not a gallbladder is diseased.

How might this improve emergency medicine practice?

Using ultrasound to identify normal anatomy and anatomic variants aids in distinguishing a diseased versus a healthy gallbladder and determining appropriate treatment.
